# Albumin‐to‐fibrinogen ratio as a promising biomarker to predict clinical outcome of non‐small cell lung cancer individuals

**DOI:** 10.1002/cam4.1428

**Published:** 2018-03-13

**Authors:** Shu‐Qi Li, Yu‐Huan Jiang, Jin Lin, Jing Zhang, Fan Sun, Qiu‐Fang Gao, Lei Zhang, Qing‐Gen Chen, Xiao‐Zhong Wang, Hou‐Qun Ying

**Affiliations:** ^1^ Department of Clinical Laboratory Jiangxi Province Key Laboratory of Laboratory Medicine The Second Affiliated Hospital of Nanchang University Nanchang 330006 Jiangxi China

**Keywords:** Albumin‐to‐fibrinogen ratio, nomogram, non‐small cell lung cancer, prognosis

## Abstract

Chronic inflammation is one of the critical causes to promote the initiation and metastasis of solid malignancies including lung cancer (LC). Here, we aimed to investigate the prognostic roles of albumin (Alb)‐to‐fibrinogen (Fib) ratio (AFR), Fib and Alb in LC and to establish a novel effective nomogram combined with AFR. Four hundred twelve LC patients diagnosed between February 2005 and December 2014 were recruited in this prospective study. The prognostic roles of AFR, Fib, Alb, neutrophil‐to‐lymphocyte ratio (NLR), platelet‐to‐lymphocyte ratio (PLR) and monocyte‐to‐lymphocyte ratio (MLR) were identified by X‐tile software, Kaplan–Meier curve, Cox regression model, and time‐dependent ROC. Pretreatment high circulating Fib, low AFR, and Alb were significantly associated with increased risk of death for LC patients, especially for non‐small cell lung cancer (NSCLC) patients in all stages. The area under curves (AUCs) of AFR, Fib, and NLR were higher than them within Alb and PLR for predicting the survival of NSCLC patients. Moreover, we found that clinical outcome of high AFR patient with chemo‐radiotherapy was superior to low AFR patient; overall survival rate of stage II‐III NSCLC patients undergoing chemo‐radiotherapy was significantly lower than the surgical patients with treatment of adjuvant chemo‐radiotherapy(*P *= 0.001) in low AFR subgroup. On the contrary, clinical outcome of the patients receiving chemo‐radiotherapy was the same to the patients undergoing surgery and adjuvant chemo‐radiotherapy (*P *= 0.405) in high AFR subgroup. In addition, c‐index of predicted nomogram including AFR (0.717) for NSCLC patients with treatment of chemo‐radiotherapy was higher than that without AFR (0.707). Our findings demonstrated that circulating pretreatment AFR might be a potential biomarker to predict clinical efficacy of surgical resection and adjuvant chemo‐radiotherapy and be a prognostic biomarker for NSCLC individuals.

## Introduction

Lung cancer (LC) is consistently ranked in the first common cancer and remains the deadliest disease worldwide 1. Although continue improvement in clinical diagnosis and treatment, the prognosis of patient with LC remains unsatisfied. Tumor‐node‐metastasis (TNM) system, histological subtype, and genetic marker were used as common prognostic tools to evaluate the overall survival (OS) 2. However, the patients harbored the same TNM stage or histological subtype showed obvious heterogeneous survival and the high cost of genetic marker restricted its application in clinic. Therefore, a simple, economical, and effective biomarker is required for precisely reflecting survival of the disease.

In recent decades, systemic inflammation has been illustrated to be an important hallmark of malignancies including LC, and it can promote initiation and metastasis as well as resistance to adjuvant chemotherapy for the disease 3. Smoking, a leading resource of chronic inflammation and reactive oxygen species in LC patients, is an vital factor to facilitate somatic genetic variation of driver and passenger genes, leading to carcinogenesis of pulmonary epithelial cells 4. Meanwhile, tumor microenvironment established by immune cell, cancer cell, and their produced cytokines triggered premetastatic niche to escape the immunological surveillance of neutrophil, monocyte and lymphocyte, and to accelerate regional or distant metastasis of the disease 5. In the same time, significant differences in circulating immune cells and inflammatory proteins such as fibrinogen(Fib), albumin(Alb), and pre‐Alb(pAlb) were commonly observed in LC patients comparing to healthy individuals, and circulating neutrophil, lymphocyte, and platelet and those proteins were candidate biomarkers to reflect the status of chronic inflammation and to evaluated prognosis of the disease 6, 7, 8. Our previous studies have indicated that the ratio of circulating inflammatory cells is superior to the single biomarker to predict the survival of colorectal and gastric cancer 9, 10. Many studies have reported the contradictory results between circulating neutrophil‐to‐lymphocyte ratio (NLR), platelet‐to‐lymphocyte ratio (PLR), and monocyte‐to‐lymphocyte ratio (MLR), and prognosis of LC 11, 12, 13. However, there is no study to investigate the association of Alb‐to‐Fib ratio (AFR) with clinical outcome of LC until now. Hence, we hypothesized that circulating AFR would be an effective biomarker to predict the survival of LC.

In present prospective study, the prognostic roles of circulating inflammatory cell and protein ratios were investigated in 412 clinical confirmed LC patients. We firstly revealed that AFR was superior to Alb, Fib, NLR, PLR, and MLR to independently predict OS in patients with LC, especially non‐small cell lung cancer (NSCLC), and it could improve predicted efficacy of the prognostic nomogram for NSCLC.

## Materials and Methods

### Eligible patient

We enrolled eligible LC patients from the Second Affiliated Hospital of Nanchang University (Jiangxi, China) and the First Affiliated Hospital of Nanchang University (Jiangxi, China) between February 2005 and January 2014 in accordance with the following included and exclusion criteria. All LC patients were newly diagnosed and classified by clinical symptom and pathological detection according to International Association for the Study of Lung Cancer (IASLC) TNM staging 14. All included patients received treatments (including surgical resection, radiotherapy or chemotherapy), which conformed to diagnostic and treatment guidelines of LC 15; of all patients, ECOG (eastern cooperative oncology group) scores were larger than two scores. On the contrary, patients with abnormal liver function, infection, inflammation‐related disease, autoimmune, hematological diseases, and other malignancies or without complete clinical and pathological data in our study were excluded. The study was approved by the Ethical Committees of the two hospitals and consent informs were signed by each included patients.

### Data collection and laboratory detection

The clinical demographic, pathological characteristics, and treatment were obtained by retrieving the medical record of each eligible individual. Two‐milliliter pretreatment circulating peripheral blood, serum, and plasma samples were collected within 7 days prior to treatment from 7:30 to 9:30 am for detection of immune cell counting, plasma Fib, and serum Alb, and automatic SYSMEX XE‐2100 hematology analyzer (Sysmex, Tokyo, Japan), SYSMEX CA‐7700 machine (Sysmex, Tokyo, Japan), and OLYMPUS AU5400 machine (Beckman Coulter, Tokyo, Japan) were used to detect those biomarkers, respectively. The inter‐ and intra‐batch variable coefficients of the three kits were less than 3.07% and 1.93%, 2.89% and 3.76%, 2.32%, and 3.15%, respectively.

### Follow‐up

After the first time therapy, all the patients were followed up the included patients every 6 months one time to obtain the survival data by means of retrieving medical record, email, and telephone, and 31 January 2017 was the deadline of the follow‐up. The 3 years’ OS was the determined endpoint in our study, and it was defined time from the first treatment to death or the deadline.

### Statistical analysis

The sample power was calculated by PASS 11 (NCSS LLC, Kaysville, UT) in our study 16. Optimum cut‐off points of each candidate inflammatory biomarkers for survival prediction were obtained using X‐tile software (http://www.tissuearray.org/rimmlab/) in our study. Chi‐square test, Mann–Whitney *U* test, and Student's *t‐*test were selected to compare the differences in qualitative and continuous variables, respectively. Kaplan–Meier curve with log‐rank test and Cox proportional hazards regression were used to determine the prognostic roles of the biomarkers in LC patients, and hazards ratio (HR) and 95% confidential interval (CI) were used to measure the strength between them. Predicted efficacy of the independent prognostic factor was evaluated and compared using time‐dependent receiver operative characteristics (ROC) curve. According to the results of Cox regression, prognostic nomogram for predicting OS within LC individuals was established and the predictive accuracy was evaluated by Harrell's concordance index (c‐index). These statistics were performed using SPSS statistical package 20.0 (SPSS, Inc, Chicago, IL) and R 3.0.3 software (Institute of Statistics and Mathematics, Vienna, Austria). All statistics were two‐sided, and *P*‐value < 0.05 was considered as statistical significance.

## Result

According to included and excluded criteria, 412 patients with LC were included in our study, and the sample power of present study reached up to 96.6% in accordance with two‐sided log‐rank test and 0.05 significant level using PASS 11.0 software. Baseline characteristics of the total LC patients and its subgroup were descripted in Table 1. Two hundred and forty‐seven and 89 patients were diagnosed as TNM stage I–III and IV NSCLC, respectively, and 58 patients were confirmed as small cell lung cancer (SCLC). Among the metastatic patients, 11, 42, 12, 13, 26, and 28 patients showed brain, bone, contralateral lung, lymphatic, malign pleural effusion, and other site metastasis, respectively. Two hundred and thirty‐one patients received surgical resection, and adjuvant chemo‐radiotherapy was carried out in 181 patients. The significant differences were found in distributions of gender, tobacco intake, hypertension, ECOG, tumor size, node metastasis, and cell differentiation in the three subgroups.

**Table 1:** Clinical and pathological characteristics in 412 eligible lung cancer patients

Variables	Categories	Total patients (*n* = 412) No. of patients (%)			
		LC patients	NSCLC (*n* = 336)		SCLC (*n* = 58)
			Stage I–III (*n* = 247)	Stage IV (*n* = 89)	
Gender*	Male	317 (76.90)	196 (79.40)	59 (66.30)	49 (84.50)
	Female	95 (23.10)	51 (20.60)	30 (33.70)	9 (15.50)
Age	Year	60.33 ± 8.97	60.42 ± 8.60	60.62 ± 9.74	58.98 ± 8.47
Tobacco**	Yes	247 (60.00)	157 (63.60)	41 (46.10)	40 (69.00)
	No	165 (40.00)	90 (36.40)	48 (53.90)	18 (31.00)
Alcohol	Yes	96 (23.30)	63 (25.50)	19 (21.30)	13 (22.40)
	No	316 (76.70)	184 (74.50)	70 (78.70)	45 (77.60)
Hypertension*	Yes	68 (16.50)	35 (14.20)	11 (12.40)	17 (29.30)
	No	344 (83.50)	212 (85.8)	78 (87.60)	41 (70.70)
Diabetes	Yes	18 (4.40)	11 (4.50)	3 (3.40)	3 (5.20)
	No	394 (95.60)	236 (95.50)	86 (96.6)	55 (94.80)
ECOG*	0	303 (73.50)	192 (77.70)	57 (64.00)	39 (67.20)
	≥1	109 (26.50)	55 (22.30)	32 (36.00)	19 (32.80)
Tumor size**	T1–T2	182 (44.20)	164 (66.40)	9 (10.10)	7 (12.10)
	T3–T4	82 (19.90)	64 (25.90)	12 (13.50)	4 (6.90)
Lymph node***	N0	122 (29.60)	116 (47.00)	4 (4.50)	1 (1.70)
	N1–N3	148 (35.90)	115 (46.60)	19 (21.30)	10 (17.20)
Differentiation***	Poor	75 (18.20)	51 (20.60)	19 (21.30)	5 (8.60)
	Well	111 (26.90)	103 (41.70)	8 (9.00)	0 (0)
Therapy	S	59 (14.30)	59 (23.89)	0 (0)	0 (0)
	SC	172 (56.10)	152 (61.45)	0 (0)	12 (20.69)
	C	181 (43.90)	36 (8.74)	89 (100)	46 (79.31)
Metastasis	Brain	11 (2.70)	N/A	10 (11.24)	1 (1.72)
	Bone	42 (10.20)	N/A	30 (33.70)	6 (1.03)
	Contralateral lung	12 (2.90)	N/A	8 (8.99)	4 (6.90)
	Lymphatic	13 (3.20)	N/A	7 (7.87)	6 (10.34)
	Malign pleural effusion	26 (6.30)	N/A	19 (21.35)	4 (6.90)
	Other	28 (6.80)	N/A	15 (16.85)	11 (18.97)
Leukocyte	*10^9^/L	6.61 (2.66–19.04)	6.54 (2.78–17.59)	7.10 (2.66–19.04)	6.80 ± 2.28
Neutrophil	*10^9^/L	4.37 (1.34–15.45)	4.24 (1.34–13.36)	4.82 (1.38–15.45)	4.15 (1.54–11.36)
Lymphocyte*	*10^9^/L	1.60 (0.19–15.43)	1.64 (0.19–15.43)	1.51 (0.43–3.50)	1.59 (0.50–3.70)
Monocyte	*10^9^/L	0.43 (0.01–1.52)	0.42 (0.01–1.52)	0.55 ± 0.28	0.50 ± 0.24
Platelet	*10^9^/L	210.00 (59.00–555.00)	218.63 ± 74.81	228.85 ± 91.17	215.00 (62.00–555.00)
Fib	mg/dL	3.81 ± 1.27	3.68 ± 1.28	3.92 (1.32–6.41)	4.04 ± 1.09
Alb*	g/L	39.17 (10.3–50.00)	39.38 (10.30–50.00)	37.93 ± 4.02	39.37 (29.45–46.83)
NLR**		2.74 (0.10–13.36)	2.61 (0.10–13.36)	3.28 (0.91–13.36)	2.77 (1.00–12.63)
PLR		136.58 (10.56–1173.68)	131.43 (10.56–1173.68)	152.20 (34.57–593.55)	137.15 (51.29–383.33)
MLR**		0.28 (0.003–1.33)	0.26 (0.003–1.14)	0.39 (0.02–1.07)	0.29 (0.08–1.33)
AFR		10.04 (4.54–34.39)	10.94 (4.54–34.39)	9.30 (5.19–27.80)	9.38 (6.01–30.63)

Abbreviation: N/A, not available; ECOG score, eastern cooperative oncology group score; Fib, fibrinogen; Alb, albumin; NLR, neutrophil‐lymphocyte ratio; PLR, platelet‐lymphocyte ratio; MLR, monocyte‐lymphocyte ratio; AFR, albumin/fibrinogen ratio. S, surgical resection without adjuvant chemo‐radiotherapy; SC, surgical resection with adjuvant chemo‐radiotherapy; C, chemo‐radiotherapy without surgery; *P < 0.05, **P < 0.01, ***P < 0.001.

**Figure 1:**
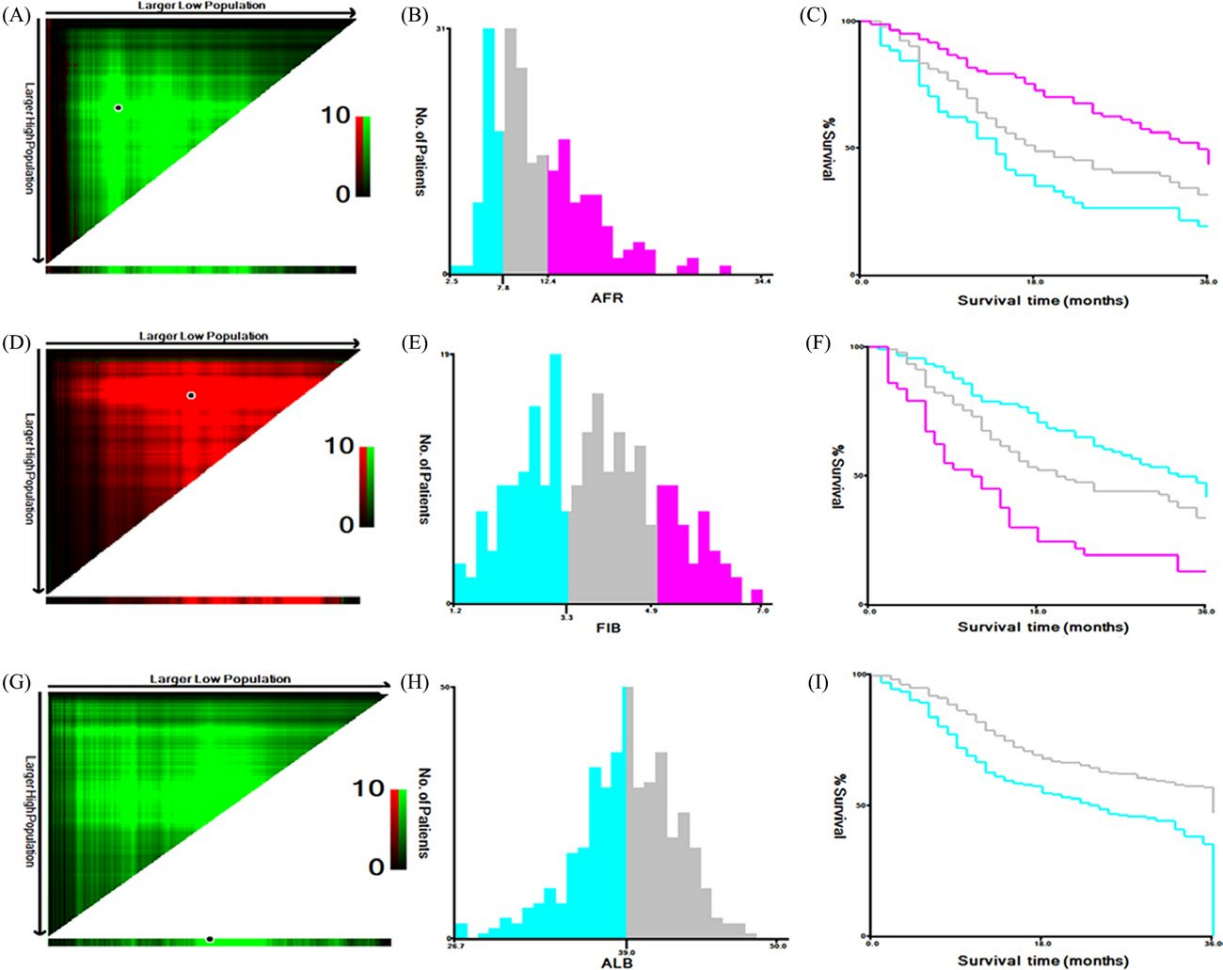
The optimal cut‐off value of preoperative circulating Alb to Fib ratio (A–C), Fib (D–F), and Alb (G–I) in 412 LC patients using X‐tile software. (A, D, G) The data were represented graphically in a right‐triangular grid where each point represents the data from a given set of divisions. The plots showed the *χ* ^2^ log‐rank values produced, dividing them into three or two groups by the cut‐off point. The optimal cut‐points (7.80, 3.30, and 39.00, respectively) were determined by locating the brightest pixel on the X‐tile plot. The distribution of number of patients was shown on the histogram (B, E, H) and corresponding populations were displayed on the Kaplan–Meier curve (C, F, I), respectively.

**Figure 2:**
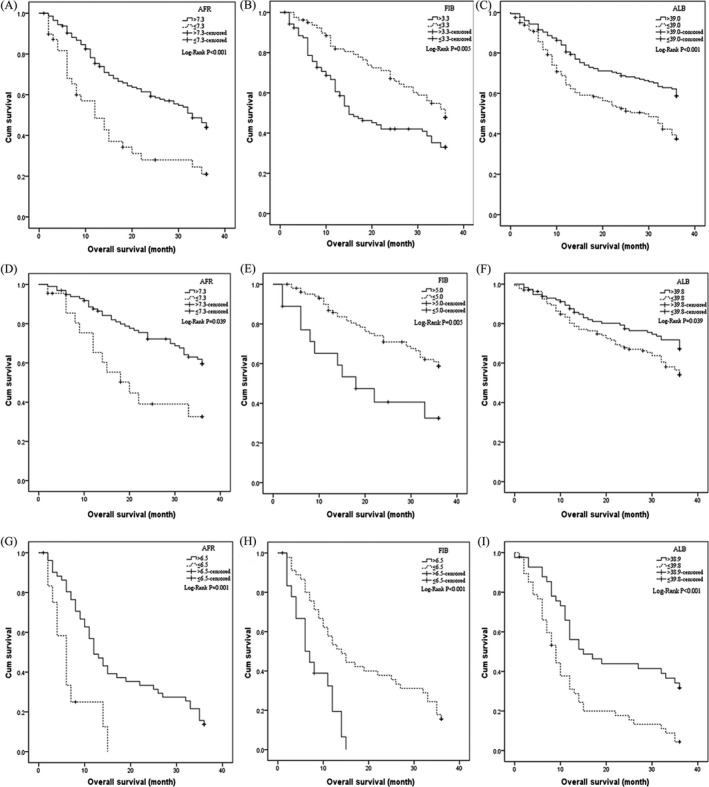
Kaplan–Meier curves of AFR, Fib, and Alb for 3 years’ OS in NSCLC patients. In NSCLC patients: (A) AFR, (B) Fib and (C) Alb; in stage I–III NSCLC subgroups: (D) AFR, (E) Fib and (F) Alb; in stage IV NSCLC subgroups: (G) AFR, (H) Fib and (I) Alb.

We detected the circulating concentrations of the candidate inflammation‐related cells and proteins, and the results were showed in Table 1. Among LC patients, circulating median or mean concentrations of AFR, NLR, PLR, MLR, Fib, and Alb were 10.04 (4.54–34.39), 2.74 (0.10–13.36), 136.58 (10.56–1173.68), 0.28 (0.003–1.33), 3.81 ± 1.27 mg/dL, 39.17 (10.30–50.00) g/L, respectively, and significant differences in NLR, MLR, and Alb were observed in the three subgroups. The respective optimal thresholds of these biomarkers in LC patients were 7.80, 2.70, 144.00, 0.20, 3.30 mg/dL, and 39.00 g/L for survival prediction in LC patients (Fig. 1). Subsequently, patients were divided into low or high groups in accordance with the best cut‐off values of these biomarkers. Significant differences of 3 years’ OS were observed in the patients harbored low and high AFR (*P *<* *0.001), Alb (*P *<* *0.001), Fib (*P *=* *0.002), NLR (*P *<* *0.001), PLR (*P *=* *0.001) and MLR (*P *=* *0.009; Fig. 2), respectively. Whereas, gender, tobacco and alcohol intakes, status of hypertension, diabetes, and ECOG were not associated with 3 years’ OS. Stage II–IV (adjusted HR = 2.31, 95% CI = 1.20–4.45 for stage II; adjusted HR =–2.52, 95% CI = 1.31–4.83 for stage III; adjusted HR = 5.26, 95% CI = 2.56–10.81 for IV stage), limited and extensive disease (adjusted HR = 3.10, 95% CI = 1.36–7.07 and adjusted HR = 5.20, 95% CI = 2.38–11.39, respectively), and only bone metastasis (adjusted HR = 1.90, 95% CI = 1.18–3.07), low AFR and Alb (adjusted HR = 1.79, 95% CI = 1.23–2.61 and adjusted HR = 1.59, 95% CI = 1.21–2.07, respectively), high NLR (adjusted HR = 1.45, 95% CI = 1.10–1.91), PLR (adjusted HR = 1.37, 95% CI = 1.05–1.79), and Fib (adjusted HR = 1.43, 95% CI = 1.00–2.05) were significantly associated with poor survival of LC. Whereas, there was no association between T_3–4_ tumor size, lymph node, poor differentiation, distant metastasis, and MLR and OS in patients with the disease (Table 2).

**Table 2:** Univariate and multivariate analyzes of Cox regression model for candidate prognostic factors for lung cancer

Variables	Univariate Cox regression			Multivariate Cox regression		
	HR	95% CI	*P*	HR	95% CI	*P*
Gender (female)	0.83	0.60–1.14	0.25			
Age (>65 years)	1.27	0.96–1.69	0.10			
Tobacco (yes)	1.17	0.90–1.54	0.25			
Alcohol (yes)	1.04	0.76–1.41	0.82			
Hypertension (yes)	1.05	0.74–1.48	0.80			
Diabetes (yes)	0.86	0.44–1.68	0.66			
ECOG (≥1)	1.22	0.91–1.62	0.18			
Therapy (chemo‐radiotherapy)	3.11	2.37–4.078	<0.001	0.78	0.58–1.04	0.09
Tumor stage						
II	2.28	1.19–4.40	0.014	2.31	1.20–4.45	0.012
III	3.07	1.64–5.75	<0.001	2.52	1.31–4.83	0.005
IV	7.38	4.01–13.58	<0.001	5.26	2.56–10.81	<0.001
Limited disease	4.02	1.83–8.82	0.001	3.10	1.36–7.07	0.007
Extensive disease	8.55	4.34–16.85	<0.001	5.20	2.38–11.39	<0.001
Tumor size (T3–T4)	1.79	1.24–2.60	0.002	1.22	0.83–1.79	0.317
Lymph node (N1–N3)	2.45	1.668–3.60	<0.001	1.57	0.99–2.49	0.054
Metastasis						
Brain metastasis	3.94	1.99–7.80	<0.001	2.03	0.95–4.33	0.068
Bone metastasis	3.63	2.47–5.35	<0.001	1.90	1.18–3.07	0.008
Contralateral lung	3.06	1.60–5.85	0.001	1.59	0.76–3.34	0.223
Lymphatic metastasis	2.08	1.09–3.96	0.027	0.10	0.47–2.71	0.995
Malign pleural effusion	3.08	1.92–4.91	<0.001	1.60	0.90–2.84	0.110
Other metastasis	3.52	2.24–5.54	<0.001	1.68	0.94–3.00	0.078
Differentiation (poor)	1.55	1.02–2.37	0.04	0.93	0.81–1.20	0.788
AFR (≤7.8)	1.97	1.36–2.85	<0.001	1.79	1.23–2.61	0.003
Fib (>3.3 mg/dL)	1.71	1.21–2.42	0.003	1.43	1.00–2.04	0.049
Alb (≤39.0 g/L)	1.66	1.27–2.16	<0.001	1.59	1.21–2.07	0.001
NLR (>2.7)	1.70	1.30–2.22	<0.001	1.45	1.10–1.91	0.008
PLR (>144.0)	1.55	1.19–2.02	0.001	1.37	1.05–1.79	0.023
MLR (>0.2)	1.51	1.10–2.07	0.010	1.18	0.86–1.64	0.308

Abbreviation: HR, hazard ratio; CI, confidence interval; AFR, albumin‐fibrinogen ratio; Fib, fibrinogen; Alb, albumin; NLR, neutrophil‐lymphocyte ratio; PLR, platelet‐lymphocyte ratio; MLR, monocyte‐lymphocyte ratio; ECOG score, eastern cooperative oncology group score; Multivariate analysis with covariant, such as gender, age, tobacco, alcohol, hypertension, diabetes, ECOG, therapy, tumor stage, and metastasis.

**Figure 3:**
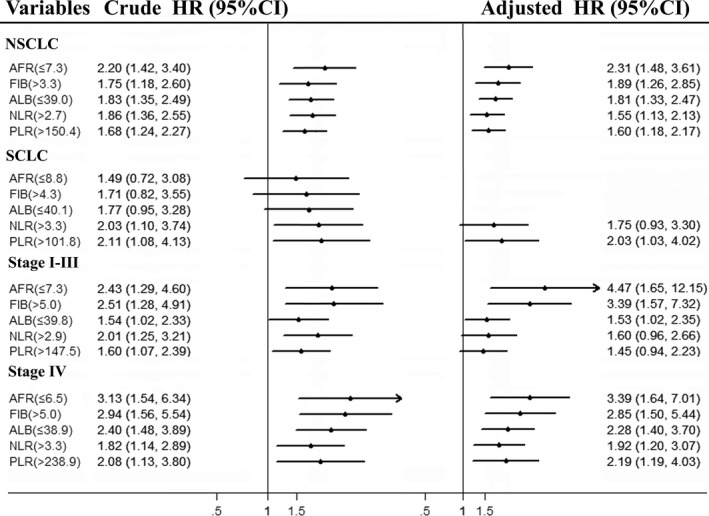
Cox regression forest plot of circulating inflammatory biomarkers in each subgroup. HR, hazard ratio; CI, confidence interval.

**Figure 4:**
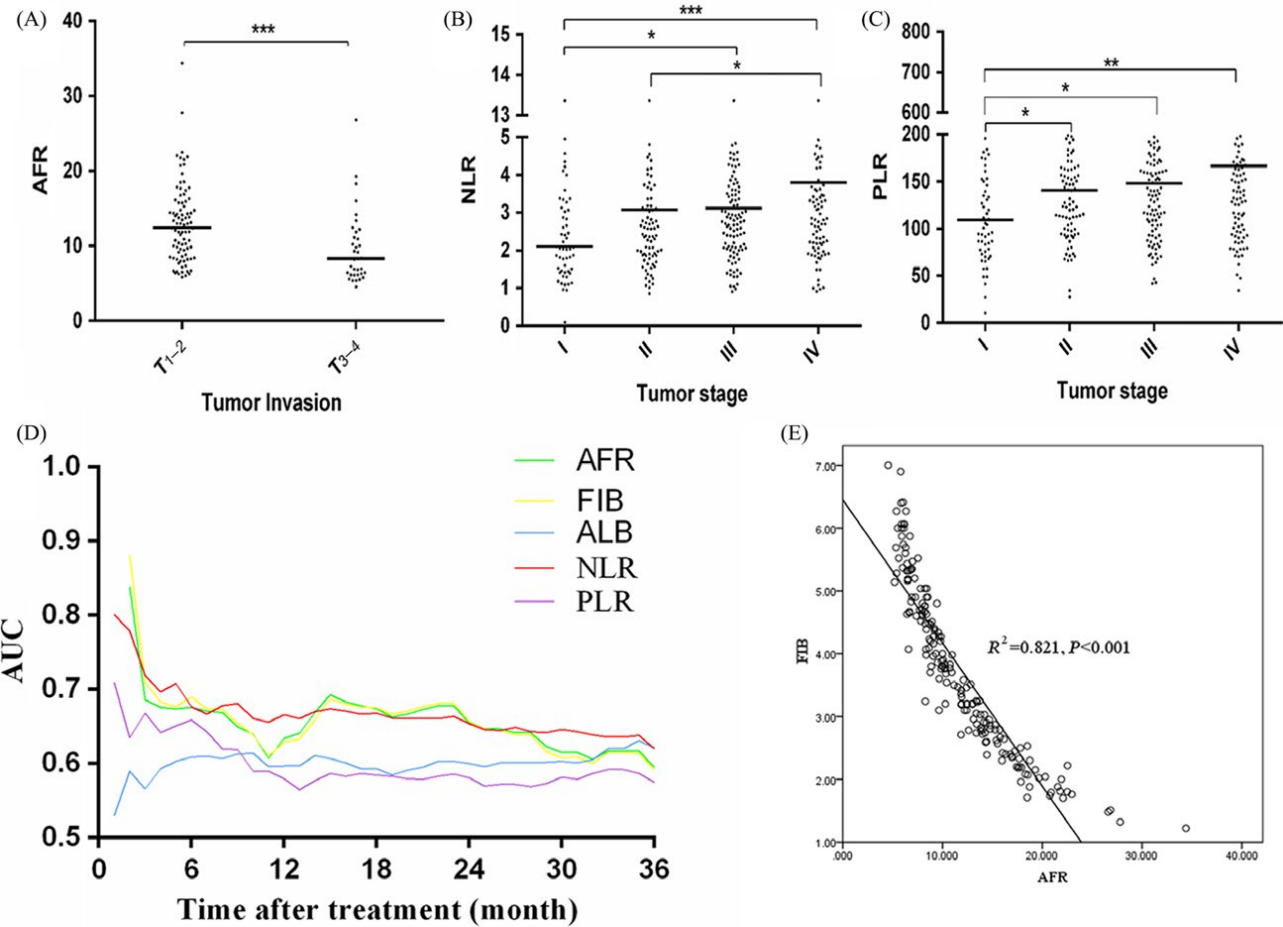
The correlation of prognostic parameters with clinical characteristics and comparison of prognostic parameters in NSCLC patients. (A) the relationship between tumor size and AFR in NSCLC; (B and C) the relationship between tumor stage and NLR and PLR in NSCLC; (D) time‐dependent ROC analysis of pretreatment circulating AFR, Fib, Alb, NLR, and PLR; (E) scatter dot presentation comparison of AFR and Fib. *P <* *0.05, **P <* *0.01, ***P <* *0.001.

To further analysis the prognostic values of AFR, Fib, Alb, NLR, and PLR in LC patients, we stratified the patients in accordance with tumor type and TNM stage. The results were showed in Figure 3. Three years’ OS within NSCLC patients harbored low AFR (adjusted HR = 2.31, 95% CI = 1.48–3.61 for overall patients; adjusted HR = 4.48, 95% CI = 1.65–12.15 for stage I–III; adjusted HR = 3.39, 95% CI= 1.64–7.01 for stage IV), Alb (adjusted HR = 1.81, 95% CI = 1.33–2.47 for overall patients; adjusted HR = 1.53, 95% CI = 1.02–2.35 for stage I–III; adjusted HR = 2.28, 95% CI = 1.40–3.70 for stage IV), and high Fib (adjusted HR = 1.89, 95% CI = 1.26–2.85 for overall patients; adjusted HR = 3.39, 95% CI = 1.57–7.32 for stage I–III; adjusted HR = 2.85, 95% CI = 1.50–5.44 for stage IV) were significantly shorter than those with high AFR, Alb and low Fib, respectively. However, circulating higher NLR (adjusted HR = 1.55, 95% CI = 1.13–2.13 for overall patients; adjusted HR = 1.92, 95% CI = 1.20–3.07 for stage IV) and PLR (adjusted HR = 1.60, 95% CI = 1.18–2.17 for overall patients; adjusted HR = 2.19, 95% CI = 1.19–4.03 for stage IV) were significantly corrected with poor clinical outcome in stage IV patients. The survival of SCLC patients harbored higher PLR (adjusted HR = 2.03, 95% CI = 1.03–4.02) was inferior to the patients with low PLR. Moreover, circulating AFR, Fib, NLR, and PLR were obviously associated with tumor size in NSCLC patients (Fig. 4A), respectively; there were significant associations between circulating NLR, PLR, and TNM stage (Table 3, Fig. 4B and C). Results of time‐dependent ROC curve showed that area under curves (AUCs) of AFR, Fib, and NLR were higher than Alb and PLR, respectively (Fig. 4D), and a significant correction was observed between AFR and Fib (*R* ^2^ = 0.821, *P *<* *0.001; Fig. 4E).

**Table 3:** Comparisons of pretreatment circulating AFR, Fib, Alb, NLR, PLR in different subgroups stratified by tobacco and TNM stage

Variables	AFR Median (95% CI)	*P* *	FIB (mg/dL) Median (95% CI)	*P* *	Alb (g/L) Median (95% CI)	*P* *	NLR Median (95% CI)	*P* *	PLR Median (95% CI)	*P* *
Tobacco										
Yes	10.33 (9.34–11.90)	0.356	3.70 (3.22–4.02)	0.512	38.91 (38.33–39.41)	0.182	2.80 (2.63–3.08)	0.308	141.94 (130.76–153.73)	0.411
No	10.22 (9.40–13.53)		3.76 (3.09–4.00)		39.27 (38.90–40.28)		2.66 (2.42–2.97)		128.41 (114.53–150.37)	
Stage										
I	12.99 (9.76–14.56)	0.171	3.20 (2.60–3.67)	0.237	39.42 (38.01–40.89)	0.045*	2.10 (1.86–2.66)	0.001*	109.16 (91.83–130.77)	0.004*
II	11.88 (10.01–14.19)		3.42 (2.81–3.79)		39.58 (38.60–40.05)		2.58 (2.31–2.82)		132.04 (117.83–150.42)	
III	9.93 (8.94–12.15)		3.94 (3.30–D4.41)		39.20 (38.82–40.00)		2.78 (2.58–3.03)		143.26 (123.81–159.10)	
IV	9.30 (8.35–12.37)		3.92 (3.20–4.46)		38.50 (37.26–39.18)		3.28 (2.81–3.69)		152.20 (132.66–167.04)	

*P < 0.05 is significant.

**Figure 5:**
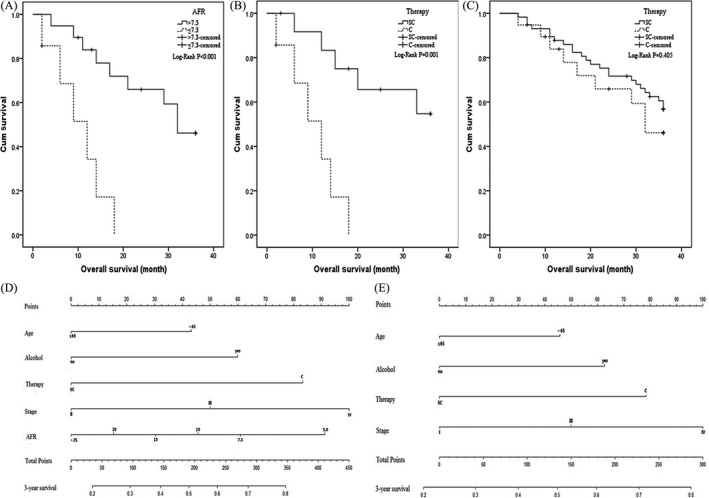
Kaplan–Meier curves of stage II–III NSCLC patients with treatment of chemo‐radiotherapy(C therapy) or combined surgical resection and chemo‐radiology (SC therapy) in low and high AFR subgroups and predicted nomogram including or without AFR for NSCLC patients. (A) Kaplan–Meier curve for overall survival probability within stage II–III NSCLC patients receiving chemo‐radiotherapy according to circulating AFR concentration; (B and C): Kaplan–Meier curve for overall survival probability within stage II–III NSCLC patients according to two therapy methods in low AFR group and high‐AFR group, respectively; (D) nomogram including AFR for predicting 3‐year OS in NSCLC patients undergoing chemo‐radiotherapy. (E) nomogram without AFR for predicting 3‐year OS in NSCLC patients undergoing chemo‐radiotherapy.

To further explore the clinical value of AFR, the association between AFR and clinical efficacy of therapeutic tool was investigated in stage II–III NSCLC patients in our study. We found that clinical outcome of high AFR patient with chemo‐radiotherapy was superior to low AFR patient (*P*‐value of log‐rank test <0.001; Fig. 5A); In low AFR subgroup, OS of the surgical patients with chemo‐radiotherapy was significantly longer than the patients undergoing chemo‐radiotherapy (*P*‐value of log‐rank test = 0.001; Fig. 5B). Whereas, no significant difference in survival was examined in high AFR patients with treatment of both surgery and chemo‐radiotherapy or only chemo‐radiotherapy (*P*‐value of log‐rank test = 0.405; Fig. 5C). Furthermore, the prognostic nomogram for NSCLC patient was established (Fig. 5D and E), and c‐indexes of the nomograms including or without AFR were 0.717 and 0.707, respectively.

## Discussion

Non‐small cell lung cancer (NSCLC) is one of the most important types of LC, and it accounts for approximately 80% of the disease 17. It is well known that environmental factors such as chronic inflammation, nutrition status and obesity and genetic factor are two main causes leading to the onset of NSCLC 18. Cross‐talking between inflammation‐related immune cells and NSCLC cells forms a cancer microenvironment to promote progression of NSCLC 19, 20. Circulating inflammatory immune cells and proteins are indicators of chronic inflammation and nutrition, and all of them may be candidate biomarkers for predicting clinical outcome of NSCLC 21.

In the present study, a prospective study including 412 LC patients was carried out to investigate the associations between pretreatment AFR, Fib, Alb, NLR, and PLR, and clinical prognosis of LC. We found that hypoalbuminemia and hyperfibrinogenemia were significantly associated with poor clinical outcome of LC, which were consistent with the reports of Gupta et al. and Allin et al. 7, 22. Circulating AFR, Fib, and Alb were significantly associated with 3‐year's OS of LC, particularly NSCLC patients. AFR and Fib were significantly related to tumor size, which was the same to the previous study 23. AUCs of AFR, Fib, and NLR were apparently higher than Alb and PLR for predicting survival of NSCLC, a significant correlation was observed between AFR and Fib, and the adjusted HR of AFR was the largest to predict the death risk of NSCLC patients in 3 years, suggesting that circulating AFR was superior biomarker to predict survival of NSCLC patients in comparison with the Fib, NLR, Alb, and PLR. Moreover, clinical outcome of high AFR stage II–III NSCLC patients undergoing chemo‐radiotherapy was significantly superior to the low AFR cases, and the survival of surgical stage II–III patients treated adjuvant chemo‐radiotherapy was longer than the cases with treatment of chemo‐radiotherapy in low AFR subgroup; whereas, the prognosis of surgical stage II–III patients with chemo‐radiotherapy was similar to chemo‐radiotherapy treated patients in high AFR subgroup. These results illustrated that AFR could predict clinical efficacy of chemo‐radiotherapy, combined surgical resection, and chemo‐radiotherapy treatment could improve the prognosis of low AFR stage II–III patients. In addition, c‐index of nomogram including AFR was higher than that without AFR, showing that AFR could improve the predictive efficacy of prognostic nomogram for chemo‐radiotherapy treated NSCLC patients.

Thus, AFR is a novel and effective biomarker to stratify the suitable NSCLC patients who appear to obviously benefit from surgical operation and adjuvant chemo‐radiotherapy and to predict the survival of NSCLC patients. The following causes might be accounted for our findings. Many transformed immune cells involved in chronic inflammation generated NSCLC‐related microenvironment, synthesized, and released numerous inflammatory factors such as fibroblast growth factor‐2 (FGF‐2), vascular endothelial growth factor (VEGF) and platelet‐derived growth factor (PDGF) 3, 6. These factors interacted with stromal cells and inflammatory cells by integrin and nonintegrin receptors to trigger the production of Fib 24, leading to high level of Fib. Plasma hyperfibrinogenemia contributed to hypercoagulability state and simultaneously promoted adhesion and survival of tumor cells after intravasation, resulting in metastatic potential in lung cancer model 25. Moreover, inflammatory cytokines such as tumor necrosis factor (TNF)‐*α* and IL‐6 suppressed synthesis of Alb, leading to hypoproteinemia in NSCLC patients 26. Additionally, previous studies have shown that hyperfibrinogenemia and hypoproteinemia are significantly related to recurrence, metastasis, and poor OS of NSCLC patients 27, 28. Furthermore, AFR, a ratio of serum Alb to plasma Fib, could amplify the sensitivity of inflammation and nutrition status in NSCLC patients and it was superior to the single Alb and Fib to predict survival of NSCLC. Importantly, AFR corresponded to the severity of inflammation could seriously predict the efficacy of chemo‐radiotherapy.

The present study is the first time to report the clinical value of AFR within NSCLC. Our study established nomogram containing AFR was an easy‐to‐use system for accurately estimating 3 years’ survival after common therapeutic tool. However, only two‐center prospective design, the heterogeneity of included patients and small sample size were the limitations in our study. Moreover, no validation cohort was included to verify our findings in our study. Therefore, a prospective cohort with multiple‐central designs and large sample size are warrant to validate the role of AFR in predicting clinical efficacy of surgical resection and adjuvant chemo‐radiotherapy and survival of NSCLC patients.

In conclusion, circulating pretreatment AFR might be a potential and economic biomarker to predict clinical efficacy of surgical resection and chemo‐radiotherapy and clinical outcome of NSCLC patients.

## Conflict of Interest

All authors declare no conflict of interest, financial in the publication of the study.
